# Effects of an exercise and manual therapy program on physical impairments, function and quality-of-life in people with osteoporotic vertebral fracture: a randomised, single-blind controlled pilot trial

**DOI:** 10.1186/1471-2474-11-36

**Published:** 2010-02-17

**Authors:** Kim L Bennell, Bernadette Matthews, Alison Greig, Andrew Briggs, Anne Kelly, Margaret Sherburn, Judy Larsen, John Wark

**Affiliations:** 1 Centre for Health, Exercise & Sports Medicine, Melbourne Physiotherapy School, University of Melbourne, Australia; 2 Department of Physical Therapy, University of British Columbia, Canada; 3 School of Physiotherapy and Curtin Health Innovation Research Institute, Curtin University of Technology, Australia; 4 Hydrotherapy Consulting and Training, Brisbane, Australia; 5 University of Melbourne Department of Medicine and Bone & Mineral Service, Royal Melbourne Hospital, Australia

## Abstract

**Background:**

This randomised, single-blind controlled pilot trial aimed to determine the effectiveness of a physiotherapy program, including exercise and manual therapy, in reducing impairments and improving physical function and health-related quality of life in people with a history of painful osteoporotic vertebral fracture.

**Methods:**

20 participants were randomly allocated to an intervention (n = 11) or control (n = 9) group. The intervention group attended individual sessions with an experienced clinician once a week for 10 weeks and performed daily home exercises with adherence monitored by a self-report diary. The control group received no treatment. Blinded assessment was conducted at baseline and 11 weeks. Questionnaires assessed self-reported changes in back pain, physical function, and health-related quality of life. Objective measures of thoracic kyphosis, back and shoulder muscle endurance (Timed Loaded Standing Test), and function (Timed Up and Go test) were also taken.

**Results:**

Compared with the control group, the intervention group showed significant reductions in pain during movement (mean difference (95% CI) -1.8 (-3.5 to -0.1)) and at rest (-2.0 (-3.8 to -0.2)) and significantly greater improvements in Qualeffo physical function (-4.8 (-9.2 to -0.5)) and the Timed Loaded Standing test (46.7 (16.1 to 77.3) secs). For the perceived change in back pain over the 10 weeks, 9/11 (82%) participants in the intervention group rated their pain as 'much better' compared with only 1/9 (11%) participants in the control group.

**Conclusion:**

Despite the modest sample size, these results support the benefits of exercise and manual therapy in the clinical management of patients with osteoporotic vertebral fractures, but need to be confirmed in a larger sample.

**Trail registration:**

NCT00638768

## Background

Vertebral fractures are common sequelae of osteoporosis often resulting in ongoing pain, musculoskeletal, respiratory and postural abnormalities, and deterioration in physical functioning and quality of life [1-5]. Of additional concern, the likelihood of sustaining further fractures increases substantially after an initial fracture [6,7] and fracture risk is higher in individuals with back muscle weakness and hyper-kyphosis of the thoracic spine [8]. This cyclical relationship between risk of vertebral fracture and physical impairments emphasizes the importance of developing effective treatments to reduce these physical risk factors for subsequent fracture, pain and physical dysfunction.

Currently, drug therapy aimed at improving the underlying osteoporosis is the primary treatment for individuals with vertebral fractures [9]. However, while antiresorptive drugs such as bisphosphonates, hormone therapy, and raloxifenes may address the specific bone deficits, the majority do not directly affect the pain and physical impairments that accompany prevalent vertebral fractures. Physiotherapy is a non-pharmacological treatment that encompasses a range of interventions including manual techniques and exercise and may play an important role in this clinical population through its potential impact on pain, impairment and physical function. However, there are no randomised controlled trials of a multimodal physiotherapy program, and a limited number of studies investigating exercise specifically in people who have sustained a vertebral fracture [10-12].

Therefore, the aim of this pilot study was to evaluate the efficacy of a physiotherapy program which incorporates manual techniques, clinician-led exercises, and home exercises designed to reduce pain, increase back extensor and lower limb muscle strength, and improve posture, trunk stability and trunk mobility. It was hypothesised that improvements in these impairments would result in better physical functioning and quality of life in people with a history of osteoporotic vertebral fracture.

## Methods

### Design

This study was a single assessor-blinded randomised controlled pilot trial. Potential participants underwent telephone screening and were required to have had a lateral spinal radiograph to diagnose vertebral fracture and dual energy X-ray absorptiometry (DXA) scan of the hip and spine to diagnose osteoporosis based on WHO criteria. Following baseline assessment, eligible participants were randomly allocated to an intervention or control group. The randomisation sequence was generated *a priori *using the random number function in Excel (Microsoft Corporation, USA) by an independent investigator not directly involved in assessment of participants. These were concealed in opaque envelopes and stored in a locked central location. The envelopes were opened sequentially at the time of intervention assignment by an administrator who informed the treating therapist by facsimilie or email.

### Participants

20 men and women (17 F, 3 M) aged over 50 years were recruited from the community via advertisements in local clubs, libraries, and the print and radio media in metropolitan Melbourne, Australia and from medical specialists.

The inclusion criteria were: (i) if female, at least five years post-menopause, defined as a cessation of menstrual cycles for ≥5 years; (ii) aged > 50 years; (iii) primary osteoporosis defined as DXA T score < -2.5 at either the spine or proximal femur with at least one painful vertebral crush or wedge fracture sustained between 3 months and 2 years previously, defined as where the anterior height was reduced by ≥20% compared with its posterior height and the posterior height of the adjacent superior or inferior vertebra [13]; (iv) stable dose of medication for treatment of osteoporosis (eg. hormone replacement therapy, bisphosphonates) for at least 6 months; (v) community dwelling and able to attend for treatment; and (vi) English speaking.

The exclusion criteria were: (i) secondary causes of bone loss such as osteomalacia, glucocorticoid medication; (ii) co-morbidities that would exclude participation in exercise such as severe heart or pulmonary disease, inflammatory joint disease, severe osteoarthritis, psychiatric condition, neuromuscular condition; (iii) acute vertebral fracture sustained within the past 3 months; (iv) radicular signs or symptoms; (v) back pain radiating into the lower limb; (vi) previous participation in a formal pain management program for back pain; (vii) physiotherapy for back pain in the past 6 months; and (viii) allergic reaction to adhesive tape or skin condition that would prevent use of tape.

Ethical approval was obtained from the University of Melbourne Human Research Ethics Committee. All participants provided written informed consent.

### Interventions

All participants were requested to refrain from seeking other forms of treatment during the 10 week trial. However, due to ethical considerations, medication was permitted as required.

#### Intervention group

Participants were treated by one of four experienced physiotherapists located at three private physiotherapy centres for 10 weekly individual sessions each lasting approximately 45 minutes. A number of centres were needed to geographically cover the metropolitan region. The treatment was standardized and the therapists were trained prior to the study to deliver the treatment. A comprehensive treatment manual and DVD were produced for the therapists and the importance of following the protocol emphasised. However, it was considered important to allow the therapists to adjust the intensity of the standardised manual treatment techniques and exercise prescription to match the participants' physical capabilities and change in clinical status: thus the dosage could be altered as required. Deletion of techniques or exercises was permitted if the therapist deemed them inappropriate. At the end of each treatment session for each participant, the physiotherapist completed a checklist to assess adherence with the standard approach.

A standardized progressive treatment protocol was devised based on the literature and clinical experience (Table 1). The aims of the physiotherapy treatment were to i) decrease back pain; ii) improve posture; iii) improve thoracic spine mobility; iv) strengthen trunk extensor and lower limb muscles; v) improve trunk control; vi) provide education. The therapist applied postural taping which was worn full-time for the first week. A protective skin barrier followed by non-rigid, hypoallergenic tape was firstly applied to provide skin protection, followed by rigid strapping tape for postural adjustments and proprioceptive feedback. The taping technique aimed to encourage a retracted scapular and pectoral girdle posture and promote thoracic spine extension. At each treatment session the therapist also performed soft tissue massage and passive accessory central posterior-anterior mobilisation techniques on the thoracic spine. Furthermore, the therapist provided education regarding the aetiology and pathophysiology of osteoporosis, the origin of the pain, the aims of treatment, the importance of patient involvement and adherence, back care, postural awareness and activities to avoid in order to foster positive self management behaviours. The therapist taught the patient exercises to be performed at home: those exercises that addressed posture and range of motion were performed daily whilst strengthening and trunk control exercises were performed three times per week. A 10 week intervention was chosen to allow sufficient time to achieve strength gains in postural muscles and increase spinal mobility [14,15].

**Table 1:** Physiotherapy and home exercise program

Technique/Exercise	Dosage	Weeks
Postural taping *	Worn full time	1
from anterior aspect of each shoulder, posteriorly and obliquely down to opposite rib cage		
Soft tissue massage *	5 mins	1-10
Performed in prone to erector spinae, rhomboids, upper trapezius -stroking, circular frictions and petrissage		
Passive accessory postero-anterior vertebral mobilisation *	5 mobilising movements at each central level × 2 reps	1-10
In prone starting at T1 down to 2 levels below the most painful vertebral region Grade 2-3 depending on level		
Supine lying over rolled up towel	5-10 mins	1
Towel placed lengthways along the back to facilitate thoracic extension		Daily
Erect sitting with transversus abdominus stabilising	10 sec hold × 5 reps	1-10
Sitting forward on a chair or stool with no back rest. Chin retraction, scapular retraction and TA contraction		Daily
Elbows back in sitting	5 sec hold × 5 reps	1-10
Hands behind head with elbows pointing out to side. Pressing elbows back by performing scapular retraction		Daily
Trunk mobility in sitting	5 reps in each direction	1-10
Hands on shoulders, gentle rotation in both directions and lateral flexion to each side		Daily
Head to wall in standing	10 sec hold × 5 reps	1-10
Back and heels against wall with rolled up towel behind head. Chin retraction		Daily
Standing corner stretch	10-30 sec hold × 3 reps	2-10
Facing corner, both hands at chest height placed on wall and moving in closer to stretch anterior chest		Daily
Walking hands up wall in standing	5 sec hold × 5 reps	3-10
Facing wall, walking hands up wall until arms upstretched then holding hands off wall		Daily
Shoulder flexion in supine	10 sec hold × 5 reps	3-10
Arms outstretched holding onto a cane/towel and taking arms over head to hold at end of range		Daily
Standing wall push ups	8-10 reps × 2	1-10
Facing wall with arms in front at shoulder height. Keeping body straight, bending and straightening elbows		3×/week
Seated row with dumbbells	8-10 reps × 2	1-10
Upright sitting and pull hands up towards chest by bending elbows and then lowering		3×/week
Seated overhead dumbbell press	8-10 reps × 2	3-10
With elbows bent and out to side, press dumbbells straight up until arms extended overhead		3×/week
Bridging in supine	5-10 sec hold × 5	1-2
Knee bent and feet flat on ground. Pushing through feet to lift back and pelvis off ground		3×/week
Hip extension in prone	8-10 reps × 2	3-10
Raising one leg off the ground and then the other		3×/week
Half squats - progress to holding dumbbells	8-10 reps × 2	1-2
Standing in front of chair and squatting down to touch chair with buttocks then standing up		3×/week
Step ups - progress to holding dumbbells	8-10 reps × 2	3-10
Stepping up and down a 10 cm step. Alternate legs		3×/week
Scapular retraction with theraband in sitting	8-10 reps × 2	1-10
Holding theraband in both hands with elbows tucked into sides and performing wrist extension, supination and shoulder external rotation then scapular retraction		3×/week
Four point kneeling with transversus abdominus	8-10 reps × 2	2
Push into floor with hands, knees and feet then draw navel up and in. Hold 5 secs		3×/week
Four point kneeling with one arm and leg lift	8-10 reps × 2	3-10
As above, then lift one arm off ground. Progress to also lifting extended leg off ground at same time		3×/week
Prone lying with arm elevation	5-10 sec hold × 5	2-3
Arms at shoulder height and bent at elbows. Scapular retraction then lift arms off floor		3×/week
Prone trunk extension	5-10 sec hold × 5	4-10
Lift head and shoulders off floor while maintaining chin retraction		3×/week

* performed by the therapist

#### Control group

Control group participants did not receive any additional intervention or complete any home exercises during the 10-week study.

### Outcome assessment

Participants were assessed at baseline and at 11 weeks by an assessor blinded to group allocation. Age, gender, number and location of the vertebral fractures, height and body mass were obtained at the baseline assessment.

A number of outcome measures were collected for this study. Overall average back pain in the week prior to assessment was self-assessed at rest and during movement by separate single, 11-point horizontal numeric rating scales with terminal descriptors of (0 = no pain; 10 = worst pain possible). Numeric rating scales have established clinimetric properties in back pain [16] and a change of at least 2 points is thought to represent a clinically meaningful improvement [17,18]. A similar scale was used to measure average amount of restriction to daily activities in the past week (0 = no restriction; 10 = maximal restriction possible). Participants also rated their perceived change in back pain over the 10 weeks (compared to baseline) on an ordinal scale (1-much worse, 2-slightly worse, 3-no change, 4-slightly better, 5-much better) [18].

Health-related quality of life was measured using one generic questionnaire and one disease-specific questionnaire. The AQoL comprises 15 items on ordinal scales with four levels per item covering five dimensions (illness, independent living, social relationships, physical senses and psychological wellbeing). It produces a single utility index that ranges from -0.04 (worst possible health-related quality of life) to 1.00 (full health-related quality of life). The AQoL has strong psychometric properties [19,20]. The Qualeffo-41 is a quality-of-life questionnaire especially developed for measuring quality of life in patients with osteoporotic vertebral deformities [20]. It consists of 41 questions arranged in five domains: pain, physical function, social function, general health perception, and mental function. Domain scores plus a total score are scaled from 0 - 100 where a lower score represents better quality of life. The Qualeffo is repeatable and discriminates well between patients with vertebral fractures and control subjects [21].

The Timed Up and Go test is a validated and reliable test of physical function in older individuals [22]. A stop watch was used to time the participant rising from a standard arm chair, walking around a cone on the floor 3 m away, returning to the chair and sitting down again [22]. The participant was barefoot and was asked to perform the task at their own pace. An explanation and demonstration was provided by the investigator but no practice trials were given. The test was performed once.

Thoracic kyphosis was measured using a Dualer Electric Inclinometer (North American Fork, Utah), and followed a previously established protocol [23]. Results of the previous study found high reproducibility (ICC [1,1] = 0.93), (95% CI = 0.66 - 0.99) of this measurement technique, and a standard error of measurement (SEM) of 2° or ± 4.3% of total thoracic kyphosis.

To assess combined trunk and arm endurance in people with vertebral osteoporosis, Shipp et al [24] developed a reliable and valid assessment called the Timed Loaded Standing test. This test measures the time a person can stand while holding a two-pound dumbbell in each hand with the arms at 90 degrees of shoulder flexion and the elbows extended.

Participant adherence was obtained by recording the number of physiotherapy sessions attended (out of a maximum number of 10). Those in the intervention group completed a daily log-book to record the number of home exercise sessions completed. Following the trial completion they were also asked to rate the effort with which they completed the home exercises as well as how frequently they followed the physiotherapist's instructions on five point scales with 1 being 'no effort' or 'never' and 5 being 'maximal effort' or 'always', respectively. Adverse events and the use of co-interventions (including other forms of exercise) were recorded for both groups in a log-book and by open-probe questioning by the assessor at trial completion.

### Sample size and data analysis

The primary outcome was average back pain during movement measured on a numeric rating scale. As this was a pilot study, a sample size calculation was not performed. All analyses were conducted on an intention-to-treat principle using all randomized participants using the Statistical Package for the Social Sciences (Norusis/SPSS Inc., Chicago IL, USA). Demographic characteristics, baseline data and adherence data were summarised by descriptive statistics. For outcomes measured using an essentially continuous scale, differences in mean change from baseline were compared between groups using general linear models (analysis of covariance) adjusting for baseline levels of the outcome measure. Measures of participant-perceived change in back pain were presented descriptively. The p value was set at p < 0.05.

## Results

**Figure 1:**
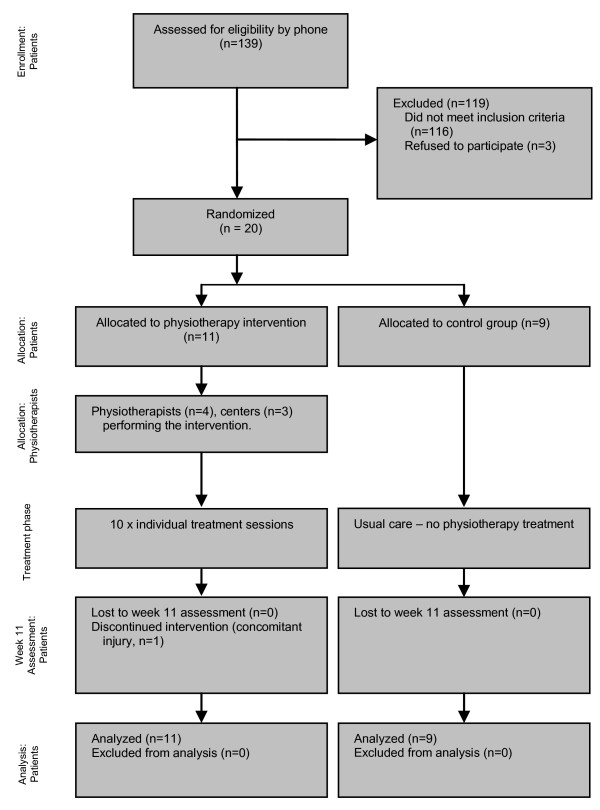
**Flow of participants through trial**.

We recruited 20 participants (11 physiotherapy, 9 control) and all completed the 10-week trial. Figure 1 shows the flow of participants through the trial. The demographic details of the participants are shown in Table 2. The ages ranged from 53 to 90 years. The majority of participants were women and the three men who took part were all randomised to the intervention group. Seventeen individuals (8 controls, 9 intervention) had a single vertebral level fracture whilst one (control) had fractures at two levels and two (intervention) had fractures at three levels. The majority of fractures occurred in the mid-lower thoracic (T7-T12) region. There were no significant baseline differences between the two groups (Table 2).

**Table 2:** Demographic information for the physiotherapy and control groups given as the mean (standard deviation) unless otherwise specified

	Physiotherapy group N = 11	Control group N = 9
Age (years)	66.2 (8.0)	66.3 (11.8)
Height (cm)	160.4 (7.7)	158.7 (4.1)
Body mass (kg)	68.1 (12.8)	68.3 (12.4)
Gender - Female (n)	7	9
Number of vertebral fractures	15	10
Number of individuals with a single vertebral fracture	9	8
Number of individuals with fractures at each level		
T4	3	0
T5	1	0
T6	2	1
T7	2	2
T8	1	4
T9	1	1
T12	2	0
L1	1	1
L2	1	1
L5	1	0

There were significant differences in the magnitude of change from baseline in the intervention group compared with the control group for a number of outcome measures (Table 3). The intervention group showed reductions in pain on movement and at rest following the 10 week treatment, while the control group reported increased pain over this time frame. These between-group differences in change in pain were significant. Similarly, the intervention group showed significantly greater improvements in the Qualeffo physical function score compared with the control group. There was no significant difference between groups for changes in other domains of the Qualeffo or in AQoL scores. For the physical impairment measures, there was a significant difference between groups in the Timed Loaded Standing test with the intervention group showing an improvement and the control group deteriorating. There was no difference in change in kyphosis or change in Timed Up and Go scores between groups.

**Table 3:** Mean (SD) of groups, mean (SD) difference within groups, and mean (95% CI) difference between groups adjusting for the baseline value of the measure, for outcomes with interval data.

Outcome	Groups				Difference within groups		Difference between groups*
			
	Week 0		Week 11		Week 11 minus Week 0		Week 11 minus Week 0
				
	Physio n = 11	Cont n = 9	Physio n = 11	Cont n = 9	Physio	Cont	Physiotherapy minus Control
Pain on movement (0-10)	3.1 (2.7)	2.1 (2.0)	1.8 (2.3)	3.1 (1.8)	-1.3 (2.4)	1.0 (1.4)	-1.8 (-3.5 to -0.1)
Pain on rest (0-10)	3.0 (2.7)	1.4 (1.8)	1.1 (2.2)	2.0 (2.6)	-1.9 (2.3)	0.6 (1.0)	-2.0 (-3.8 to -0.2)
Restriction (0-10)	2.5 (2.0)	2.7 (2.6)	1.3 (2.4)	3.2 (2.9)	-1.2 (2.4)	0.6 (2.0)	-1.8 (-3.9 to 0.3)
AQol (-0.04 - 1.00)	0.51 (0.25)	0.56 (0.14)	0.54 (0.25)	0.49 (0.16)	0.03 (0.17)	-0.08 (0.11)	0.10 (-0.04 to 0.24)
Qualeffo Total (0-100)	29 (10)	26 (7)	21 (11)	27 (8)	-7.2 (11.2)	1.2 (4.1)	-7.1 (-14.9 to 0.8)
Qualeffo Pain (0-100)	51 (24)	32 (20)	29 (30)	37 (19)	-22.7 (28.8)	5.0 (11.5)	-19.3 (-41.5 to 3.0)
Qualeffo Physical function (0-100)	16 (8)	15 (9)	11 (6)	15 (8)	-5.4 (6.2)	-0.2 (4.1)	-4.8 (-9.2 to -0.5)
Qualeffo Social function (0-100)	31 (17)	35 (12)	23 (16)	33 (11)	-7.9 (13.8)	-1.2 (4.8)	-8.0 (-17.3 to 1.4)
Qualeffo General health (0-100)	49 (14)	43 (13)	39 (25)	45 (16)	-9.9 (20.0)	1.9 (8.1)	-12.1 (-27.9 to 3.7)
Qualeffo Mental function (0-100)	32 (10)	30 (7)	30 (13)	32 (10)	-1.3 (13.6)	2.2 (7.2)	-2.8 (-13.2 to 7.7)
Thoracic kyphosis (deg)	59 (9)	58 (12)	56 (10)	58 (12)	-3.2 (5.9)	-0.2 (3.7)	-2.9 (-7.9 to 2.1)
Timed up and go (s)	9.7 (2.7)	9.8 (1.7)	9.0 (1.5)	9.6 (1.7)	-0.7 (1.6)	-0.2 (3.7)	-0.5 (-1.6 to 0.6)
Timed loaded standing test (s)	50 (39)	62 (49)	82 (44)	46 (50)	32.6 (34.1)	-16.7 (29.8)	46.7 (16.1 to 77.3)

* adjusted for the baseline value of the measurement

For the perceived change in back pain over the 10 weeks, 9/11 (82%) participants in the intervention group rated their pain as 'much better' while 1/11 (9%) rated it as 'no change' and 1/11 (9%) rated it as 'much worse'. In contrast, 5/9 (56%) participants in the control group rated their pain as 'no change' with 1/11 (11%) participants rating it in each of the other categories.

Adherence to treatment was excellent. Eight of the 11 participants (73%) in the intervention group attended all 10 treatment sessions with the other three attending four, eight and nine sessions. Home exercise adherence ranged from 34% to 100% with the median (interquartile range) being 95% (78-100%). The home exercises were completed at a median (IQ range) intensity of 4 (3-4) whilst the extent to which the participants followed the physiotherapist's instructions was rated as 5 ('always') by 9/11 (82%) participants.

Six of the intervention group participants (55%) reported adverse events associated with treatment. These were minor and comprised increased shoulder pain (n = 2), flare-up of a wrist injury (n = 1), sore knee (n = 1) and a sore waist (n = 1) with particular exercises as well as irritation with the tape (n = 1). All of these adverse complaints settled after changing or eliminating the aggravating activity. None of the intervention group reported any co-interventions. One participant in the control group received three sessions of physiotherapy due to an increase in back pain. Four control group participants (44%) and four intervention group participants (36%) took medication for pain ranging from 1-14 days in both groups.

## Discussion

This single-blind randomised controlled pilot trial found that a 10 week physiotherapy program improved pain, function and physical impairments in people with a history of painful osteoporotic vertebral fracture sustained between 3 months and 2 years previously. To our knowledge, this study is the first RCT to investigate a *multimodal *physiotherapy program in this patient population. The results support the benefits of combined manual therapy and exercise in the clinical management of patients with osteoporotic vertebral fractures. Moreover, the program was well accepted by participants with 73% attending all physiotherapy sessions and 82% always following instructions from their treating clinician.

Acute pain following a vertebral fracture often settles within a few weeks although many individuals experience chronic pain with the risk of pain generally increasing with the number and severity of vertebral fractures [25]. In fact, a recent case series of 107 consecutive patients found that 80% still had pronounced pain one year after the fracture [26]. In our study, pain during movement and at rest was reduced by 42% and 63% respectively in the intervention group whilst the control group showed increases in pain of 48% and 43%. The difference in change in rest pain between groups was of an amount that is considered clinically meaningful (2 points) while the difference in change in movement pain between groups approached this clinically meaningful amount [17]. The benefits were also confirmed by the results from the Qualeffo pain subscale and by the participants' overall rating of change in back pain. No major adverse events were reported by participants in the intervention group, suggesting that the manual therapy and exercise interventions were safe.

Lower quality of life has also been reported in individuals with vertebral fracture [27] and the mean (SD) AQoL score for our sample of 0.53 (0.20) is much lower than the comparable Australian population mean for 60-69 year olds of 0.79 (0.19) [28]. Despite reduced health-related quality of life in our sample, there was no significant improvement following the intervention as measured by the total Qualeffo score or the AQoL although trends were noted. Significant benefits were seen in the physical function domain of the Qualeffo which is not surprising given that the treatment focused on techniques to address physical impairments. Psychological interventions such as cognitive behavioural therapy may be needed to reduce psychological impairments that impact on quality of life such as anxiety, fear and depression, consistent with the biopsychosocial model of treating chronic musculoskeletal pain.

The intervention aimed to reduce thoracic kyphosis because of the relationship between the magnitude of kyphosis and spinal loads [29]. Patients with vertebral fracture have been found to have a greater kyphosis than their age-matched counterparts [30-32] and higher spinal loads as a consequence [33]. This may contribute to the well-documented increased risk of subsequent fractures after an individual has sustained an initial vertebral fracture. In addition to the mechanical loading implications of thoracic kyphosis, functional implications include limitations in pulmonary function, compromised balance and therefore increased falls risk, and exacerbation of back extensor muscle weakness [32]. These highlight a biomechanical rationale for treatment modalities aimed at reducing kyphosis.

While it is recognised that there will be a degree of fixed structural thoracic kyphosis [34], often times the kyphosis is compounded by habitually-poor posture and weakness of the back extensor muscles. Given a potentially-modifiable component to the kyphosis, our intervention program incorporated postural retraining, and exercises to improve range of thoracic extension and strength of the back extensor and posterior shoulder musculature. Postural taping was worn full-time for the first week to provide patients with the sensation of improved posture to facilitate postural retraining. In another study we found that postural tape led to an immediate 5% reduction in thoracic kyphosis in 15 patients with vertebral fracture [23]. Given that there were no associated changes in the surface electromyographic activity of the trunk muscles, the reduction in kyphosis was likely to have been achieved passively through mechanical support from the tape and activation of deep postural muscles. In the current study, a similar reduction (5%) in thoracic kyphosis was found following treatment in the intervention group compared to no change in the control group. This difference was larger than the standard error of measurement of ± 2 degrees or ± 4.3% [23] but the result did not reach statistical significance probably due to the small sample size. It is also possible that a longer time frame is needed to yield larger effects: one case series showed that an orthotic brace worn over 6 months led to an 11% reduction in kyphosis [35].

Our strengthening exercises concentrated specifically on the back extensor and posterior trunk postural muscles in order to promote a more neutral spinal posture and minimise deleterious flexion moments [29,33]. The exercises were of low intensity in order to minimise compression loads through already-weakened vertebrae and to target slow twitch muscle fibres which predominate these muscle groups [36]. We did not use a maximum strength test for the back extensor muscles because of the potential risk of further vertebral fracture. Instead we used the Timed Loaded Standing test to indicate combined trunk and arm muscle endurance [24]. The intervention group showed a 65% improvement in holding time whilst the control group showed a 26% reduction. This suggests that the intervention was effective in increasing muscle endurance although part of the improvement may also be related to a reduction in back pain. Similarly, performance of a single low-intensity back extensor exercise in a group of postmenopausal women with osteoporosis, some of whom had vertebral fractures, significantly improved back extensor strength as well as quality-of-life over a 4 month period [37]. We have also found altered neuromuscular patterns of paraspinal muscle activity in individuals who have sustained vertebral fractures compared to those without fracture [38]. This suggests that in addition to strengthening, specific neuromuscular retraining might be effective.

Our program included manual therapy that involved mobilising techniques applied gently to the thoracic spine. Application of central postero-anterior force (Maitland-mobilisation) to thoracic spinous processes causes extension of the thoracic motion segments [39], leading to improved range of movement into extension locally and at adjacent motion segments [40,41]. Ultimately, manual therapy of this nature may improve active extension range and reduce pain related to intervertebral stiffness. Whilst a survey of Canadian physiotherapists revealed that manual therapy is used by 45% of therapists in the management of patients with osteoporosis, over 91% had concerns about its safety particularly with regards to causing vertebral or rib fractures [42]. However, a study designed to investigate the safety of spinal mobilization showed that the in vivo loads applied by therapists during these techniques were well below in vitro fracture loads, suggesting a reasonable safety margin [39]. We also had no adverse effects related to the manual therapy components of our program.

Given that our intervention was multimodal, it is not possible to establish which of the individual treatment components was more or less effective or which contributed to each of the outcomes observed. It is also possible that a mechanism underlying part of the improvements in the physiotherapy group relates to the therapeutic environment including interaction with the therapist rather than the interventions *per se*. Given that our control group received no treatment rather than placebo physiotherapy, this cannot be ascertained. However, the placebo effect has been found to be apparent for pain but less so for other physical measurements [43]. Thus, the improvements noted for the other measurements such as the Timed Loaded Standing test are more likely to be due to the specific techniques and exercises.

There are few randomised controlled trials investigating the effectiveness of conservative, non-pharmacological interventions applied in the chronic phase to the osteoporotic population with vertebral fractures. Malmros et al [11] found that a 10-week exercise program that focused on balance, strength and lumbar stabilisation improved balance and level of daily function and decreased pain and use of analgesics. This study also demonstrated improvement in the quality of life of participants even beyond the active training period. Another study investigating a 6-month minimally-supervised home-based exercise program comprising stretching, strengthening and walking also improved quality of life with benefits sustained at 12 months [12]. Gold et al [10] found that in 185 older women (mean age of 81 years), group exercise and coping classes for six months delivered by a physiotherapist and social worker led to improvements in back extensor strength and psychological impairments but not in pain levels. That pain was not reduced may relate to the fact that only 40% of the participants reported pain in the previous month. Our results offer preliminary evidence for the efficacy of rehabilitative interventions delivered after a symptomatic osteoporotic vertebral fracture. The efficacy of these interventions for reducing the risk of incident fracture should now be explored.

Our study has several limitations. First, the most obvious is the small sample size. However despite this, significant differences in outcomes were found between the intervention and control groups. These results need to be confirmed in a larger sample. Furthermore we did not adjust the analyses for multiple comparisons which increases the risk of making a Type 1 error. Nevertheless, the results were consistent across several measurement instruments. Second is that neither the participants nor the care providers were blinded which may exaggerate the estimates of treatment effects [44]. However, there is some debate in the literature about whether it is appropriate to use a placebo treatment for interventions such as physiotherapy where it is difficult to isolate the direct and indirect effects of the therapy [45]. It has been argued that these effects are unlikely to be distinct, additive and divisible and that using a placebo-controlled trial design will not detect the whole treatment effect and may in fact generate false negative results. Third, as we did not include a follow up assessment, compliance with unsupervised home exercises and maintenance of benefits over time are unknown. Fourth, whether our physiotherapy program reduces the risk of future fracture would need to be tested in a larger cohort over a longer period. Such a benefit was noted in a prospective 10 year follow up study where a back extensor strengthening program was effective in reducing the risk of subsequent vertebral fracture in post menopausal women [46]. Fifth, as participants were unable to consistently identify a mechanism of injury for their vertebral fracture(s), consistent with the stochastic nature of vertebral fractures [47], we are unable to judge the efficacy of the intervention program relative to the fracture mechanism. Finally, the majority of fractures in our sample were located in the thoracic region. Given evidence that lumbar fractures are associated with more severe pain and lower quality-of-life than thoracic fractures [48,49], the results may not be directly generalisable to lumbar fractures.

## Conclusions

This randomised controlled trial found that a 10-week program of physiotherapy was effective in reducing pain and improving physical function and back muscle endurance in a group of 19 individuals with a history of painful vertebral fracture. Despite the modest sample size, these results support the benefits of exercise and manual therapy in the clinical management of patients with osteoporotic vertebral fractures, but need to be confirmed in a larger sample.

## Competing interests

The authors declare that they have no competing interests.

## Authors' contributions

All authors read and approved the final manuscript. KB conceived of the study and participated in its design. She was the overall co-ordinator of the study and developed the physiotherapy treatment, trained the therapists, performed the statistical analyses and wrote the first draft of the paper. BM assisted with recruitment, training of physiotherapists and performed the outcome assessments. AG assisted with recruitment, testing and contributed to the writing of the manuscript. AB assisted with recruitment and contributed to the writing of the manuscript. AK assisted with recruitment and co-ordination of the study. MS was involved with the design of the study, developed the physiotherapy treatment and assisted with training of therapists. JL was involved with the design of study and development of the treatment protocol. JW was involved with the design of the study and drafting the manuscript. All authors read and approve the final paper.

## Pre-publication history

The pre-publication history for this paper can be accessed here:

http://www.biomedcentral.com/1471-2474/11/36/prepub
